# Supplementary material for: The Thermo-Oxidative Behavior of Cotton Coated with an Intumescent Flame Retardant Glycine-Derived Polyamidoamine: A Multi-Technique Study
Source: Polymers (Basel). 2021 Dec 14;13(24):4382. doi: 10.3390/polym13244382 (PMC8707849; doi:10.3390/polym13244382)
Supplement: Supplementary file 1 [file polymers-13-04382-s001.zip › polymers-1492706-supplementary.pdf]

Supporting Information

The Thermo-Oxidative Behavior of Cotton Coated with an Intumescent Flame Retardant Glycine-Derived Polyamidoamine: A Multi-Technique Study

Claudia Forte,[1*] Jenny Alongi,[2] Alessandro Beduini,[2] Silvia Borsacchi,[1] Lucia Calucci,[1] Federico Carosio,[3] Paolo Ferruti,[2] Elisabetta Ranucci[2]

[1] Institute of the Chemistry of OrganoMetallic Compounds (ICCOM), National Research Council, via G. Moruzzi 1, 56124 Pisa, Italy; claudia.forte@pi.iccom.cnr.it (C.F.); silvia.borsacchi@pi.iccom.cnr.it (S.B.); lucia.calucci@pi.iccom.cnr.it (L.C.)

[2] Dipartimento di Chimica, Università degli Studi di Milano, via C. Golgi 19, 20133 Milano, Italy jenny.alongi@unimi.it (J.A.); alessandro.beduini@unimi.it (A.B.); paolo.ferruti@unimi.it (P.F); elisabetta.ranucci@unimi.it (E.R.)

[3] Dipartimento di Scienza Applicata e Tecnologia, Politecnico di Torino, Alessandria campus, viale T. Michel, 15121 Alessandria, Italy; federico.carosio@polito.it (F.C.)

* Correspondence: claudia.forte@pi.iccom.cnr.it (C.F.); Tel.: +39–050-315.2462

S. Dipolar dephasing ^{13}C CP-MAS experiments

In order to assess the degree of condensation of the aromatic structures in the chars obtained by heating COT and COT/M-GLY in air, the CP-MAS pulse sequence with dipolar dephasing [1, 2, 3] was applied. In this experiment, a delay period, during which the ^{1}H decoupler is turned off, is inserted between the CP contact period and ^{13}C signal acquisition. During the delay, the magnetization of carbons with strong dipolar couplings with protons, i.e. carbons bonded or close in space to protons, is attenuated by dephasing. By lengthening the delay duration, the magnetization of carbons more bonds removed from hydrogens is progressively selected.

Differential dephasing was first investigated on the model compound Emodin with three substituted fused planar rings (Figure S3). The obtained data, shown in Figure S3a, yielded decay rates that decrease with increasing the number of bonds between a given carbon and protons. The decaying trends of the ^{13}C signals *vs* the dipolar dephasing period recorded for the standard compound can in principle be considered as calibration curves for converting the dephasing rates into estimated distances.

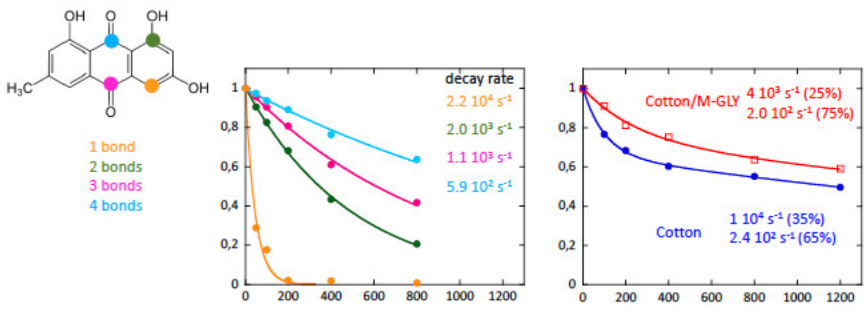

Figure S1. Molecular structure of the model compound Emodin. Trends of the intensity of the signals in the ^{13}C CP-MAS spectra recorded with dipolar dephasing as a function of the dephasing delay duration for the indicated carbons of Emodin and for the aromatic carbons (110-160 ppm) of COT (blue)

and COT/M-GLY (red) treated at 420 °C. Points represent experimental data; lines indicate best fittings to exponential or bi-exponential functions as explained in the text.

When performed on chars of COT and COT/M-GLY heated to 420 °C, dipolar dephasing experiments gave the decaying trends shown in Figure S1b for the global signal arising from aromatic carbons (110-160 ppm). In both cases, a bi-exponential behavior was observed, with a faster decaying component with a rate of $4{\cdot}10^3$ s^{-1} for COT/M-GLY and of 10^4 s^{-1} for COT and a slower component with a rate of about $2{\cdot}10^2$ s^{-1} for both samples. The bi-exponential behavior is most probably due to the presence of polyaromatic structures with different condensation degree in the chars, which hampers a direct conversion of the decaying rates into distances. However, data clearly indicate that COT/M-GLY, showing a slower decay at all dipolar dephasing delays, forms a char containing aromatic clusters of average larger size with respect to COT.

References

1. Opella, S.J.; Frey, M.H. Selection of nonprotonated carbon resonances in solid-state nuclear magnetic resonance. *J. Am. Chem. Soc.* **1979**, *101*, 5854-5856. DOI: 10.1021/ja00513a079.
2. Alemany, L.B.; Grant, D.M.; Alger, T.D.; Pugmire, R.J. Cross polarization and magic angle sample spinning NMR spectra of model organic compounds. 3. Effect of the carbon-13-proton dipolar interaction on cross polarization and carbon-proton dephasing. *J. Am. Chem. Soc.* **1983**, *105*, 6697-6704. DOI: 10.1021/ja00360a025.
3. Mao, J.-D.; Schmidt-Rohr, K. Recoupled long-range C–H dipolar dephasing in solid-state NMR, and its use for spectral selection of fused aromatic rings. *J. Magn. Reson.* **2003**, *162*, 217-227. DOI: 0.1016/S1090-7807(03)00012-0.
